# Supplementary material for: Meta-Analysis and Gene Set Enrichment Relative to ER Status Reveal Elevated Activity of MYC and E2F in the “Basal” Breast Cancer Subgroup
Source: PLoS One. 2009 Mar 9;4(3):e4710. doi: 10.1371/journal.pone.0004710 (PMC2650420; doi:10.1371/journal.pone.0004710)
Supplement: Dataset S1 — GSEA results from first screen. The file contains a zipped archive of the HTML results of the second GSEA screen. Reports for enriched or depleted datasets may be accessed from the “index.html” link within the my_analysis.GseaPreranked.1219975950625.rpt.zip ZIP archive. Enrichment plots are available for each of the 50 most enriched and depleted gene sets. (13.32 MB ZIP) [file pone.0004710.s001.zip › my_analysis.GseaPreranked.1219975950625.rpt.zip!//BREASTCA_TWO_CLASSES.html]

Details for gene set BREASTCA\_TWO\_CLASSES[GSEA]

|  || Dataset | zSco.ERneg.to.ERpos\_collapsed |
| Phenotype | NoPhenotypeAvailable |
| Upregulated in class | na\_pos |
| GeneSet | BREASTCA\_TWO\_CLASSES |
| Enrichment Score (ES) | 0.52772415 |
| Normalized Enrichment Score (NES) | 2.249247 |
| Nominal p-value | 0.0 |
| FDR q-value | 0.0 |
| FWER p-Value | 0.0 |
Table: GSEA Results Summary

  

Fig 1: Enrichment plot: BREASTCA\_TWO\_CLASSES      
 Profile of the Running ES Score & Positions of GeneSet Members on the Rank Ordered List

  

| PROBE | GENE SYMBOL | GENE\_TITLE | RANK IN GENE LIST | RANK METRIC SCORE | RUNNING ES | CORE ENRICHMENT || 1 | E2F3 |  |  | 6 | 7.369 | 0.0177 | Yes |
| 2 | SLC43A3 |  |  | 10 | 7.126 | 0.0351 | Yes |
| 3 | LPIN1 |  |  | 15 | 6.951 | 0.0519 | Yes |
| 4 | MSN |  |  | 20 | 6.766 | 0.0683 | Yes |
| 5 | FOXM1 |  |  | 21 | 6.760 | 0.0850 | Yes |
| 6 | CRYAB |  |  | 39 | 6.465 | 0.0997 | Yes |
| 7 | CCNA2 |  |  | 52 | 6.297 | 0.1143 | Yes |
| 8 | ZCCHC11 |  |  | 77 | 6.026 | 0.1274 | Yes |
| 9 | YES1 |  |  | 91 | 5.947 | 0.1411 | Yes |
| 10 | BTG3 |  |  | 102 | 5.850 | 0.1547 | Yes |
| 11 | CTPS |  |  | 123 | 5.706 | 0.1673 | Yes |
| 12 | VLDLR |  |  | 184 | 5.400 | 0.1761 | Yes |
| 13 | PSIP1 |  |  | 204 | 5.307 | 0.1877 | Yes |
| 14 | RDX |  |  | 207 | 5.298 | 0.2007 | Yes |
| 15 | NUP93 |  |  | 210 | 5.281 | 0.2135 | Yes |
| 16 | TP53BP2 |  |  | 245 | 5.119 | 0.2236 | Yes |
| 17 | CHEK1 |  |  | 253 | 5.088 | 0.2356 | Yes |
| 18 | PDCD5 |  |  | 270 | 5.025 | 0.2468 | Yes |
| 19 | MCM7 |  |  | 289 | 4.970 | 0.2577 | Yes |
| 20 | ADSL |  |  | 298 | 4.943 | 0.2693 | Yes |
| 21 | CSDA |  |  | 337 | 4.835 | 0.2784 | Yes |
| 22 | ITGB8 |  |  | 354 | 4.777 | 0.2889 | Yes |
| 23 | C10ORF7 |  |  | 375 | 4.736 | 0.2991 | Yes |
| 24 | GABRP |  |  | 412 | 4.649 | 0.3079 | Yes |
| 25 | GART |  |  | 415 | 4.639 | 0.3192 | Yes |
| 26 | CKS1B |  |  | 416 | 4.638 | 0.3306 | Yes |
| 27 | SNRPD1 |  |  | 440 | 4.552 | 0.3401 | Yes |
| 28 | KIAA0020 |  |  | 467 | 4.469 | 0.3491 | Yes |
| 29 | MAD2L1 |  |  | 477 | 4.446 | 0.3594 | Yes |
| 30 | PARD3 |  |  | 482 | 4.401 | 0.3700 | Yes |
| 31 | POLR2F |  |  | 509 | 4.331 | 0.3787 | Yes |
| 32 | DLG7 |  |  | 521 | 4.306 | 0.3885 | Yes |
| 33 | PPP1CB |  |  | 533 | 4.274 | 0.3982 | Yes |
| 34 | XRCC6 |  |  | 581 | 4.186 | 0.4050 | Yes |
| 35 | TUBB |  |  | 582 | 4.183 | 0.4153 | Yes |
| 36 | NCAPD3 |  |  | 661 | 4.016 | 0.4193 | Yes |
| 37 | CEP57 |  |  | 739 | 3.862 | 0.4230 | Yes |
| 38 | SERBP1 |  |  | 742 | 3.856 | 0.4324 | Yes |
| 39 | MSH2 |  |  | 819 | 3.748 | 0.4359 | Yes |
| 40 | AOF2 |  |  | 854 | 3.701 | 0.4424 | Yes |
| 41 | GDI2 |  |  | 886 | 3.648 | 0.4491 | Yes |
| 42 | NUP155 |  |  | 977 | 3.517 | 0.4509 | Yes |
| 43 | IARS |  |  | 987 | 3.501 | 0.4589 | Yes |
| 44 | HMGN3 |  |  | 1005 | 3.478 | 0.4662 | Yes |
| 45 | MYC |  |  | 1025 | 3.447 | 0.4732 | Yes |
| 46 | PFKP |  |  | 1165 | 3.281 | 0.4708 | Yes |
| 47 | RSU1 |  |  | 1252 | 3.188 | 0.4721 | Yes |
| 48 | OPRS1 |  |  | 1255 | 3.185 | 0.4799 | Yes |
| 49 | MSH6 |  |  | 1276 | 3.164 | 0.4861 | Yes |
| 50 | KIAA0090 |  |  | 1297 | 3.140 | 0.4924 | Yes |
| 51 | ZNF22 |  |  | 1327 | 3.108 | 0.4979 | Yes |
| 52 | PCNA |  |  | 1346 | 3.087 | 0.5041 | Yes |
| 53 | OGT |  |  | 1387 | 3.052 | 0.5086 | Yes |
| 54 | DERA |  |  | 1469 | 2.979 | 0.5098 | Yes |
| 55 | GMPS |  |  | 1543 | 2.907 | 0.5115 | Yes |
| 56 | ILF2 |  |  | 1644 | 2.807 | 0.5108 | Yes |
| 57 | SF3A3 |  |  | 1676 | 2.779 | 0.5153 | Yes |
| 58 | FKBP5 |  |  | 1729 | 2.743 | 0.5181 | Yes |
| 59 | ELAVL1 |  |  | 1751 | 2.727 | 0.5233 | Yes |
| 60 | IKZF1 |  |  | 1788 | 2.703 | 0.5272 | Yes |
| 61 | HP1BP3 |  |  | 1868 | 2.632 | 0.5277 | Yes |
| 62 | NARG1 |  |  | 2377 | 2.271 | 0.4948 | No |
| 63 | EIF2C2 |  |  | 2384 | 2.269 | 0.5000 | No |
| 64 | CDK4 |  |  | 2546 | 2.176 | 0.4931 | No |
| 65 | PARP1 |  |  | 2571 | 2.161 | 0.4966 | No |
| 66 | IRF1 |  |  | 2636 | 2.127 | 0.4970 | No |
| 67 | HADHA |  |  | 2701 | 2.090 | 0.4973 | No |
| 68 | RAB9 |  |  | 2819 | 2.030 | 0.4935 | No |
| 69 | EPHB4 |  |  | 2836 | 2.023 | 0.4973 | No |
| 70 | DDT |  |  | 2977 | 1.942 | 0.4914 | No |
| 71 | CDKN2C |  |  | 3148 | 1.866 | 0.4832 | No |
| 72 | MTHFD1 |  |  | 3258 | 1.814 | 0.4794 | No |
| 73 | GNAS |  |  | 3395 | 1.751 | 0.4734 | No |
| 74 | MAPK1 |  |  | 3460 | 1.726 | 0.4728 | No |
| 75 | PDGFB |  |  | 3540 | 1.690 | 0.4710 | No |
| 76 | ST13 |  |  | 4254 | 1.412 | 0.4204 | No |
| 77 | SFRS4 |  |  | 4477 | 1.327 | 0.4068 | No |
| 78 | GNA12 |  |  | 4829 | 1.211 | 0.3832 | No |
| 79 | ITGB1BP1 |  |  | 4830 | 1.211 | 0.3862 | No |
| 80 | THOC1 |  |  | 5299 | 1.048 | 0.3533 | No |
| 81 | ZFP36L1 |  |  | 5368 | 1.023 | 0.3507 | No |
| 82 | CUL2 |  |  | 5535 | 0.963 | 0.3405 | No |
| 83 | KIF5B |  |  | 5668 | 0.924 | 0.3327 | No |
| 84 | DPM1 |  |  | 5904 | 0.847 | 0.3170 | No |
| 85 | STAM |  |  | 6423 | 0.674 | 0.2794 | No |
| 86 | TUBGCP3 |  |  | 6561 | 0.631 | 0.2706 | No |
| 87 | THRAP4 |  |  | 7127 | 0.444 | 0.2288 | No |
| 88 | CREB1 |  |  | 7221 | 0.412 | 0.2228 | No |
| 89 | CTNNB1 |  |  | 7241 | 0.405 | 0.2224 | No |
| 90 | TAF1 |  |  | 7305 | 0.381 | 0.2185 | No |
| 91 | CBL |  |  | 7535 | 0.295 | 0.2019 | No |
| 92 | SDHA |  |  | 7721 | 0.223 | 0.1884 | No |
| 93 | PHYH |  |  | 7788 | 0.193 | 0.1839 | No |
| 94 | CTNNA1 |  |  | 8309 | -0.001 | 0.1445 | No |
| 95 | ACTR1A |  |  | 8505 | -0.079 | 0.1299 | No |
| 96 | SERPINE1 |  |  | 8947 | -0.283 | 0.0971 | No |
| 97 | KRT8 |  |  | 8956 | -0.291 | 0.0972 | No |
| 98 | OAZ1 |  |  | 9277 | -0.446 | 0.0741 | No |
| 99 | CTGF |  |  | 9358 | -0.487 | 0.0692 | No |
| 100 | ATN1 |  |  | 9388 | -0.500 | 0.0683 | No |
| 101 | ERBB2 |  |  | 9498 | -0.560 | 0.0614 | No |
| 102 | GRB2 |  |  | 9586 | -0.610 | 0.0563 | No |
| 103 | CALM2 |  |  | 9592 | -0.613 | 0.0574 | No |
| 104 | SMAD2 |  |  | 9791 | -0.719 | 0.0442 | No |
| 105 | FGFR4 |  |  | 9832 | -0.745 | 0.0430 | No |
| 106 | PXN |  |  | 10080 | -0.895 | 0.0265 | No |
| 107 | FGFR3 |  |  | 10244 | -0.985 | 0.0165 | No |
| 108 | DEAF1 |  |  | 10455 | -1.127 | 0.0034 | No |
| 109 | SPHAR |  |  | 10801 | -1.387 | -0.0193 | No |
| 110 | HDAC3 |  |  | 10972 | -1.548 | -0.0284 | No |
| 111 | SOD1 |  |  | 11102 | -1.664 | -0.0341 | No |
| 112 | SLC35B1 |  |  | 11337 | -1.865 | -0.0472 | No |
| 113 | RPA3 |  |  | 11357 | -1.892 | -0.0440 | No |
| 114 | MLLT3 |  |  | 11472 | -2.006 | -0.0477 | No |
| 115 | CRAT |  |  | 11548 | -2.086 | -0.0482 | No |
| 116 | NIFUN |  |  | 12124 | -2.732 | -0.0851 | No |
| 117 | GPX4 |  |  | 12424 | -3.141 | -0.1000 | No |
| 118 | TOB1 |  |  | 12612 | -3.453 | -0.1056 | No |
| 119 | LRPAP1 |  |  | 12709 | -3.635 | -0.1039 | No |
| 120 | UBE2B |  |  | 12747 | -3.735 | -0.0975 | No |
| 121 | ALCAM |  |  | 12765 | -3.783 | -0.0895 | No |
| 122 | COX6C |  |  | 12819 | -3.930 | -0.0838 | No |
| 123 | CD63 |  |  | 12872 | -4.071 | -0.0777 | No |
| 124 | MNAT1 |  |  | 12891 | -4.114 | -0.0689 | No |
| 125 | TMEM127 |  |  | 13012 | -4.496 | -0.0669 | No |
| 126 | CCND1 |  |  | 13160 | -5.098 | -0.0655 | No |
| 127 | FAH |  |  | 13211 | -5.503 | -0.0557 | No |
| 128 | VAV3 |  |  | 13265 | -6.141 | -0.0445 | No |
| 129 | ATP6AP1 |  |  | 13285 | -6.357 | -0.0303 | No |
| 130 | WWP1 |  |  | 13289 | -6.419 | -0.0147 | No |
| 131 | ENPP1 |  |  | 13307 | -6.873 | 0.0010 | No |
Table: GSEA details [plain text format]

  

Fig 2: BREASTCA\_TWO\_CLASSES: Random ES distribution      
 Gene set null distribution of ES for **BREASTCA\_TWO\_CLASSES**
